# Roles of Vitamin A Metabolism in the Development of Hepatic Insulin Resistance

**DOI:** 10.1155/2013/534972

**Published:** 2013-09-30

**Authors:** Guoxun Chen

**Affiliations:** Department of Nutrition, University of Tennessee at Knoxville, Knoxville, TN 37996, USA

## Abstract

The increase in the number of people with obesity- and noninsulin-dependent diabetes mellitus has become a major public health concern. Insulin resistance is a common feature closely associated with human obesity and diabetes. Insulin regulates metabolism, at least in part, *via* the control of the expression of the hepatic genes involved in glucose and fatty acid metabolism. Insulin resistance is always associated with profound changes of the expression of hepatic genes for glucose and lipid metabolism. As an essential micronutrient, vitamin A (VA) is needed in a variety of physiological functions. The active metablite of VA, retinoic acid (RA), regulates the expression of genes through the activation of transcription factors bound to the RA-responsive elements in the promoters of RA-targeted genes. Recently, retinoids have been proposed to play roles in glucose and lipid metabolism and energy homeostasis. This paper summarizes the recent progresses in our understanding of VA metabolism in the liver and of the potential transcription factors mediating RA responses. These transcription factors are the retinoic acid receptor, the retinoid X receptor, the hepatocyte nuclear factor 4*α*, the chicken ovalbumin upstream promoter-transcription factor II, and the peroxisome proliferator-activated receptor *β*/*δ*. This paper also summarizes the effects of VA status and RA treatments on the glucose and lipid metabolism in vivo and the effects of retinoid treatments on the expression of insulin-regulated genes involved in the glucose and fatty acid metabolism in the primary hepatocytes. I discuss the roles of RA production in the development of insulin resistance in hepatocytes and proposes a mechanism by which RA production may contribute to hepatic insulin resistance. Given the large amount of information and progresses regarding the physiological functions of VA, this paper mainly focuses on the findings in the liver and hepatocytes and only mentions the relative findings in other tissues and cells.

## 1. Introduction of Vitamin A (VA)

### 1.1. The Discovery of VA

Dietary energy and nutrients are required for the survival of an individual. Diets have been considered as nutriments, medicines, and poisons for thousands of years. With the development of modern nutrition, the roles of each dietary component in health and diseases have been gradually revealed after the understanding of its chemical structures and metabolism. This has contributed enormously to the prevention and treatment of diseases related to nutrition abnormalities. However, the roles of micronutrients in the development of chronic metabolic diseases such as obesity and diabetes have not been clearly defined.

When normal growth of experimental animals was the readout, scientists began to realize that dietary factors other than proteins, carbohydrates, pure fats, and minerals were essential as well [1]. Lipid- and water-soluble vitamins started to be recognized and identified after purified diets with defined components were used to determine essential factors to support the growth of lab animals [2]. VA was the first lipid-soluble factor being observed and described [3, 4]. When rats, after weaning, were fed a synthetic diet with fat stripped of VA for eight weeks, their growth stopped. The somatic growth resumed when VA was added back to the diet [3, 4]. Since then, additional characterizations of VA have gradually revealed its roles in the general health of a subject and its use to treat diseases [5]. 

 It has been known now since then VA (retinol) and molecules with similar physiological activities are essential micronutrients for a variety of physiological functions such as vision, embryogenesis, immunity, and differentiation [6]. The development of VA deficiency will cover clinical symptoms ranging from night blindness to the increase of mortality in patients with measles [7]. On the other hand, excessive intake of VA from dietary sources or supplements has been considered to be teratogenic [8]. In addition, when patients with acne are treated with retinoic acid (RA) drugs such as isotretinoin (13-*cis *RA), a significant portion of them develop hypertriglyceridemia, an undesired side effect [9, 10]. Patients with acute promyelocytic leukaemia (APL) treated with RA gain body weight [11]. Moreover, excessive *β*-carotene intake from supplements can also cause detrimental effects [12]. All of these observations demonstrate the broad spectrum of VA's physiological functions.

### 1.2. The Sources and Storage of VA

Since humans and mammals do not synthesize VA, its nutritional need has to be satisfied through dietary intake. Dietary sources of VA exist in two forms: preformed VA (retinol or retinyl esters) and provitamin A carotenoids [13]. Preformed VA is mainly in the form of retinyl esters (REs) from animal sources. In the small intestine lumen, the released REs from digested animal products are enzymatically hydrolyzed into retinol and free fatty acids (FFAs) probably by pancreatic lipases, esterases, and intestinal phospholipases before being absorbed into enterocytes with other lipids [14]. Inside the enterocytes, a portion of retinol is reesterified to REs by the lecithin-retinol acyltransferase (LRAT) or acyl-CoA: retinol-acyltransferase (ARAT). REs are packed into chylomicrons with other dietary lipids for the delivery to the rest parts of the body *via* lymph circulation. A small portion of the retinol is directly transported *via* portal circulation [15]. REs in the plasma and the liver mainly contain the fatty acyl moieties of palmitate and stearates, regardless of the composition of fatty acid (FA) in the diet. When triglyceride (TG) is stripped away from the chylomicrons by lipoprotein lipase (LPL), REs are still associated with chylomicron remnants which are eventually taken into hepatocytes. In hepatocytes, REs are hydrolyzed into retinol and FFAs again. Retinol can be released into the blood circulation or catabolized into retinal, RA, and other metabolites for usage or disposal. In addition, retinol is reesterified into RE again and stored in stellate cells inside the liver [6].

Provitamin A molecules are from plant sources. They are found in colored fruits and vegetables and are named carotenoids such as *β*-carotene, *α*-carotene, and *β*-cryptoxanthin [16]. Recently, with the development of transgenic techniques, enzymes for the synthesis of *β*-carotene have been genetically engineered into the rice genome [16]. The produced rice containing significant amount of *β*-carotene is called golden rice. In theory, this rice can be used to provide provitamin A to populations with low dietary VA availability. Carotenoids are converted into retinal and then retinol in enterocytes and hepatocytes. The conversion of provitamin A carotenoids to retinal has two pathways. The central cleavage is mediated by *β*,*β*-carotene-15,15′-dioxygenase. The eccentric cleavage is mediated by *β*,*β*-carotene-9′,10′-oxygenase. The resulting retinal is reduced to form retinol [15, 17].

 REs stored in hepatic stellate cells can be released into hepatocytes again in case of insufficient amount of dietary VA or provitamin A intake. In hepatocytes, retinol binds to retinol-binding proteins (RBPs) to form holo-RBPs, which are released into the blood circulation. In plasma, the holo-RBPs interact with transthyretin (TTR), which binds to thyroxine (T4). This complex transports both retinol and T4. Peripheral tissues express RBP receptors such as STRA6 for the uptake of retinol from plasma [18]. Hepatocytes express RBP4 receptor-2 (RBPR2), which has been considered to be responsible for plasma retinol homeostasis [19].

### 1.3. VA Metabolism

VA homeostasis is regulated by a network of enzymes and proteins involved in the transport, production, and catabolism of retinoids [20]. VA's physiological functions are mainly mediated by its metabolites, retinal, and RA. This is achieved *via* a series of enzymes catalyzing the conversions [21]. Two oxidation steps occur during the conversion from retinol to retinal (retinaldehyde) and then from retinal to RA [22]. Retinol is reversibly converted into retinal, and retinal is irreversibly converted into RA [23]. Retinol is reversibly oxidized into retinal by two families of enzymes: alcohol dehydrogenases (ADHs) and retinol dehydrogenases (RDHs) or short-chain dehydrogenases/reductases (SDRs). Two RDHs (RDH2 and RDH10) play major roles in this step in different tissues [24, 25]. The produced retinal plays important roles in physiology. For example, vision is mediated by 11-*cis* retinal conjugated to rhodopsin in response to photon activation. Recently, retinal has been considered an antagonist for the activation of peroxisome proliferator-activated receptor *γ* (PPAR*γ*). When the level of retinaldehyde (retinal) was induced in insulin-resistant *ob/ob *mice after the deletion of *Raldh1 *gene, their insulin sensitivity was improved [26]. 

Retinal is irreversibly oxidized into RA by retinaldehyde (aldehyde) dehydrogenases (RALDHs or ALDHs) [21, 27, 28]. Currently, four RALDHs (RALDH1, RALDH2, RALDH3, and RALDH4) have been cloned and thought to be responsible for all-*trans* or 9-*cis* RA production in various tissues [29–32]. They have been collectedly called retinoid dehydrogenases [21]. RALDH1–4 proteins were observed in the mouse liver based on immunohistochemistry results, and the expression of RALDH1 was found in lipid-engorged cells [30]. The expression of *Raldh1* (also known as *Aldh1a1*) mRNA was detected weakly in the rat liver [29]. RALDH1 appears to be the predominant enzyme for RA biosynthesis [33], and elevated RA is observed to control its biosynthesis by downregulating RALDH1 through modulation of retinoic acid receptor a (RAR*α*) and the CCAAT/enhancer-binding protein *β* (C/EBP*β*) [34, 35]. In rat anterior pituitary cells, estrogen receptor alpha can regulate the expression of *Raldh1* [36].

The expression of RALDH2 and RALDH3, but not RALDH1, can be detected in the developing anterior pituitary glands of rats [37]. The expression of *Raldh4* (also known as *Aldh8a1*) mRNA was expressed at high level in the mouse liver [30]. Other potential pathways for the RA formation include oxidation of retinal by microsomal cytochrome P450 and direct production of RA by cleavage of *β*-carotene in a process that might not involve retinol or retinal as intermediates [38].

In hepatocytes, RA can be further modified by enzymes such as cytochrome P450 26A1 (CYP26A1) to more hydrophilic products [39–44]. The expression of *Cyp26a1* mRNA is rapidly induced by RA treatment and is often used as an indicator of RA production [45].

Other products derived from retinoid metabolism have been reported. Retroretinoids are a class of retinol derivatives in which the polyene tail is rigidly attached to the *β*-ionone ring by a double bond with the remaining double bonds retaining the conjugated system and extending it to the 3, 4 double bond within the ring. Another bioactive retinoid is 3, 4-dihydroretinol, also known as vitamin A2. It is abundant in freshwater fish, where it is metabolized into 11-*cis*-dehydroretinol which can serve as a ligand for visual pigments. Oxidized retinol metabolites, such as 4-oxo-retinoic acid, were reported to be highly active in determining digit development and activation of retinoic acid receptor *β* (RAR*β*). Some proteins are modified by covalent retinoylation [13].

### 1.4. Potential Transcription Factors That Mediate RA Effects: RAR, Retinoid X Receptor (RXR), PPAR*β*/*δ*, Hepatocyte Nuclear Factor 4*α* (HNF4*α*), and Chicken Ovalbumin Upstream Promoter-Transcription Factors (COUP-TFs)

#### 1.4.1. Retinoic Acid-Responsive Element (RARE)

It has been generally believed that the active metabolite of VA, RA, regulates gene expression through activation of two members of nuclear receptors [46]: RARs and RXRs [27, 28]. RAR/RXR heterodimer or RXR/RXR homodimer associated with RARE in the promoters activates the transcription of RA-responsive genes upon ligand binding [47, 48].

RA regulates gene expression through transcription factors bound to RAREs. A typical RARE contains two hexameric motifs, 5′-(A/G)G(G/T)TCA-3′, arranged as palindromes, direct repeats (DRs), or inverted repeats (IRs) [49]. These two motifs are separated by nucleotides. The common DRs with 1, 2, or 5 nucleotide spacing are termed DR1, DR2, and DR5 elements, respectively. These different DRs may determine the regulatory features of the RA-targeted genes. When bound to DR2 and DR5 elements, the 5′ half-site is occupied by RXR and the 3′ half-site by RAR [47]. On the other hand, the upstream half-site of a DR1 can be recognized by a RAR, a setting that may recruit repressor complexes to suppress gene expression. When bound to DR1 elements, the polarity of the RAR/RXR heterodimer is inverted, and the complex is unresponsive to RA stimulation, probably due to the inability of RAR ligands to induce the dissociation of corepressors [50]. RXRs can also bind as homodimers to DR1 elements and respond to *9-cis* RA. By contrast, for DR2/DR5 elements, a RAR occupies the downstream halves of these RAREs, and the complex functions as transcriptional activator. Additional arrangement of two or three hexameric motifs with variable spacing has also been identified [51]. RARE can be found even in the 3′ of a gene [52].

#### 1.4.2. RARs and RXRs for Mediating RA Responses

The major functions of VA are mediated by its active metabolite, RA. It is generally believed that all-*trans* and 9-*cis* RAs are major isoforms that mediate the regulation of gene expression [6]. However, the most important ligand is probably all-*trans* RA as the detection of 9-*cis* RA produced in a physiological setting has been challenging [6]. The RA homeostases is controlled by the expression of the enzymes responsible for its production and disposal [53]. It is worth noting that some physiological functions of retinol, such as in vision, could not be replaced by RA treatment [54].

RARs and RXRs are members in the nuclear receptor superfamily [6, 47, 55]. Nuclear receptors are transcription factors that mediate a complex array of extracellular signals into transcriptional responses in a ligand-dependent manner [46]. Other members in this family include transcription factors that bind to a variety of physiological active molecules such as endocrine steroids, vitamin D, thyroid hormone, and a large number of “orphan” receptors, whose ligands, target genes, and physiological functions were initially unknown [46, 56]. Upon binding to the ligands, nuclear receptors undergo conformational changes which allow them to interact with transcriptional cofactors and, in turn, regulate the expression of their target genes [46, 57].

RARs and RXRs are widely expressed in metabolic active tissues [58]. There are three isoforms each for RARs (RAR*α*, -*β*, and -*γ*) that bind and respond to all-*trans* and 9-*cis* RAs, and for RXRs (RXR*α*, -*β*, and -*γ*) which can only bind and respond to 9-*cis* RA [55]. Functional studies have indicated that RXR/RAR heterodimers act as the main functional units transducing RA signals in development, which needs the formation of specific heterodimers such as RXR*α*/RAR*α*, RXR*α*/RAR*β*, and RXR*α*/RAR*γ*. RAR/RXR heterodimers and RXR/RXR homodimers modulate gene expression by binding to RAREs located in the regulatory regions of their target genes [6, 47, 55]. 

Other than RARs, many nuclear receptors form heterodimers with RXRs for binding to their cognate-responsive elements, such as thyroid hormone receptors (TRs), vitamin D3 receptors (VDRs), and PPARs [46]. So far, RXRs are considered binding partners for other nuclear receptor pathways. The activation states of RXRs differ among these heterodimers and seem to depend on the nature of their partners and the binding elements. For example, in the case of RAR/RXR heterodimer, either one can be transcriptionally active. However, the ligand-bound RXR is not active unless its RAR partner binds to a ligand [59]. Either PPARs or RXRs in a PPAR/RXR heterodimer can bind to their agonists and activate transcription. The presence of both ligands results in synergistic activation. Liver X receptor (LXR)/RXR heterodimer retains *9-cis* RA responsiveness, indicating that RXR is active upon ligand binding [46]. By contrast, the TR/RXR and VDR/RXR heterodimers are thought to be nonpermissive, as they are activated by the TR ligand triiodothyronine (T3) and VDR ligand 1,25-dihydroxy-VD3 (calcitriol), respectively, but not by RXR-specific ligands. It is generally believed that, in a nonpermissive heterodimer, RXR is incapable of binding to its ligands, and thus it is often referred to as a silent partner. However, recent data indicated that RXR was able to bind to ligands and lead to dissociation of corepressors from TR, thus modulating heterodimer activity [60].

#### 1.4.3. HNF4*α* for Mediating RA Responses

Originally, HNF-4*α* (NR2A1, gene *Nr2a1*) was identified as a transcription factor enriched in liver nuclear extract and was responsible for transthyretin gene transcription [61]. HNF4*α* is a highly conserved member of the nuclear receptor superfamily. HNF4*α* binds to DNA as a homodimer and acts as a positive transcriptional regulator of many hepatic genes. Expression of *Nr2a1* gene is driven by two distinct promoters: the P1 promoter that drives expression of splice variants HNF4*α*1-6 in the liver, kidney, and intestine/colon and the P2 promoter that drives expression of splice variants HNF4*α*7-9 in the intestine/colon, stomach, and *β*-cells of the pancreas. A nonsense mutation (Q268X) in exon 7 of the *Nr2a1* gene that caused a deletion of 187 C-terminal amino acids of the HNF4*α* protein was indentified in patients with maturity-onset diabetes of the young type I (MODY1), an autosomal dominant, early-onset form of noninsulin-dependent diabetes mellitus (NIDDM) [62]. This shortened HNF4*α* protein lacks transcriptional activity and fails to dimerize and bind to DNA [63]. Another mutation due to a 2 bp deletion in exon 3 of the HNF4*α* gene which results in a truncation of the *Nr2a1* protein to 122 instead of 465 amino acids causes the carriers to have significantly lower levels of plasma TG and apolipoprotein CIII (apoCIII) than normal subjects [64].

The roles of HNF4*α* have been studied extensively. Liver-specific knockout of HNF4*α* resulted in the reduction of plasma levels of TG and cholesterol, accumulation of hepatic lipid contents, and decrease of hepatic expression levels of apolipoproteins AII, AIV, CII, and CIII [65]. Moreover, it has been shown that HNF4*α* can activate the expression of hepatic glucokinase gene (*Gck*) after it binds to the *Gck* promoter [66].

It seems that there is an interaction of PPAR*α* and HNF4*α* signaling pathways. In the promoter of mouse glycogen synthase 2 gene (*Gys2*), there are two putative PPAR response elements (PPREs): one in the upstream promoter and one in intron 1 [67]. The DR1 PPRE in the upstream promoter region is the response element for HNF4*α*. The expression level of *Gys2* mRNA in the liver of *Nr2a1*−/− mice is dramatically lower than that of the wild-type controls. In addition, the *Gys2* mRNA level in the liver of *Ppara*−/− mice is also lower than that of the wild-type control mice. PPAR*α* ligand Wy14643 no longer induces *Gys2* mRNA expression in primary hepatocytes of *Ppara*−/− mice. In Hep2G cells, PPAR*α* activation significantly reduced HNF4*α*-dependent transactivation of *Gys2* promoter, demonstrating the interaction of their signaling pathways [67].

HNF4*α*, although initially believed to be an orphan receptor, activity can be modulated by fatty acyl-coenzyme A (CoA) thioesters [68], and also by protein kinase A-mediated phosphorylation [69]. For drosophila, HNF4 (dHNF4) regulates lipid mobilization and *β*-oxidation [70]. Mutant larvae with dHnf4 deletion were unable to efficiently mobilize stored fat for energy during starvation, consistent with reduced expression of genes that control lipid catabolism and *β*-oxidation. It seems that FAs released from TGs can activate dHNF4 in fasted drosophila, which in turn drives FA oxidation for energy production [70]. This suggests that HNF4*α* may be responsive to dietary signals and important in the control of metabolic status.

It has been shown that HNF4*α* is responsible for the functions of pancreatic *β*-cells. The pancreatic *β*-cell-specific *Nr2a1*−/− knockout mice have impaired glucose-stimulated insulin secretion [71]. HNF4*α* activates the insulin gene expression through indirect and direct mechanisms [72]. HNF4*α* also regulates the expression of other pancreatic *β*-cell genes involved in glucose metabolism and nutrient-induced insulin secretion, including glucose transporter-2 and liver-type pyruvate kinase [73].

A relationship between RA signaling pathway and HNF4*α* activation has been indicated in the regulation of the hepatic gene expression. Multiple sites in the promoter of human apoCIII (*APOC3*) are responsible for positive and negative regulation of its transcription in HepG2 cells, and the proximal sequence of a 13-nucleotide element for the positive regulation of the *APOC3* transcription is identical between human and rat genomic sequences [74]. It has been shown that different proteins or forms of the same protein in the nuclear extract from HepG2 and Hela cells bound to the same 13-nucleotide sequence element in the promoter of the *APOC3 *gene [75]. The activity of this element is not affected by its orientation in a reporter construct [75]. The proximal hormone-responsive element B (−87/−72) binds strongly to HNF4, ARP1/COUP-TFII, EAR2/COUP-TFIII, EAR3/COUP-TFI, and RXR*α*/RAR*α* heterodimers, and less efficiently to homodimers of RAR*α* and heterodimers of RXR*α* with TR*β* or PPAR*α* [76]. In Hep3B cells, the treatment of RA caused the reduction of *Nr2a1* mRNA [77] and HNF4*α* protein levels after the RA treatment for 3 days [78].

It was found that RA-mediated downregulation of *α*-fetoprotein gene is dependent on the inhibition of HNF1 and HNF4*α* in Hep3B cells [77]. Since there is no RARE at the *Nr2a1* gene promoter, the mechanism for RA-mediated inhibition of HNF4*α* remains to be investigated. On the other hand, HNF4*α* regulates retinoid metabolism by activating the transcription of CRBPII gene [79]. Furthermore, it has been suggested that HNF4*α* and RXR*α* compete for occupancy of the same site in erythropoietin gene (*Epo*) promoter sequentially regulating its expression during embryogenesis [80]. In the development of mouse embryo, the hepatic expression of erythropoietin is sequentially regulated by RARs and HNF4*α* at days E10.25 and E12.25, respectively [80]. This DR2 RARE is in the 3′ hypoxia-response enhancer of *Epo* gene and is responsible for RA-mediated induction of *Epo* gene in P19 and F9 embryonal carcinomas but not in Hep3B cells [81].

#### 1.4.4. COUP-TFII for Mediating RA Responses

Chicken ovalbumin upstream promoter-transcription factors (COUP-TFs) are members of a family of evolutionarily conserved orphan nuclear receptor without known physiological ligand. Currently, this family has three members: COUP-TFI/NR2F1/ErbA-related protein-3 (EAR3), COUP-TFII/NR2F2/apolipoprotein-AI regulatory protein-1 (ARP1), and COUP-TFIII/NR2F6 (EAR2) [82]. COUP-TFs are conservative among all species, with the ligand-binding domains of COUP-TFI or -II being identical in vertebrates.

The cloning and analysis of COUP-TFII (gene, *NR2F2*) cDNA from Hela cells indicate that it is a member of nuclear receptor superfamily [83]. COUP-TFII seems to be required earlier in the development processes than COUP-TFI. COUP-TFII has been thought to suppress gene expression [84]. It has been shown to function in a variety of biological processes, such as development, cellular differentiation, growth, and metabolic homeostasis [85]. Deletion of *Nr2f2* in mouse is lethal [82]. It has been shown that mice with homozygous deletion of *Nr2f2* die at around E10 probably due to severe hemorrhage and edema, and only one-third of the heterozygote mice survive before weaning [86]. *Nr2f2* heterozygous pups have growth retardation in comparison with their wild-type controls [87]. Adult *Nr2f2* heterozygous knockout mice have lower body weight and basal plasma insulin level but higher insulin sensitivity than that of their wild-type controls [85]. They also exhibit resistance to high-fat diet- (HFD-) induced obesity and improved glucose tolerance [85].

COUP-TFII expression can be detected in a variety of metabolic active tissues and organs [88]. Since heterozygous mice with *Nr2f2* deletion have improved glucose homeostasis and increased energy expenditure, this indicates that COUP-TFII also plays roles in white adipose tissues development and energy metabolism [85]. COUP-TFII may suppress adipogenesis *via* inhibiting the expression of genes for adipocyte differentiation, such as the expression of sterol regulatory element-binding protein-1c (SREBP1c), PPAR*γ*, and CCAAT/enhancer-binding protein *α* (C/EBP*α*) [89]. COUP-TFII regulates the expression of insulin gene and several other genes involved in glucose and lipid metabolism in pancreatic *β*-cells. Heterozygous mice with *Nr2f2* deletion in pancreatic *β*-cells have impaired glucose sensitivity and abnormal insulin secretion [90].

COUP-TFII regulates gene expression *via* both protein-protein and protein-DNA interactions [76]. For example, COUP-TFII binds to hormone response elements (HREs) recognized by other nuclear receptors and, in turn, modulates the expression of these genes. It has been shown that COUP-TFII can bind to a variety of HREs that contain direct or inverted imperfect AGGTCA repeats with various spaces. COUP-TFII can also interact with the common partners or general transcriptional factors to modulate gene expression [84, 91]. For example, the amount of RXR available for the formation of high-affinity DNA-binding complexes of the thyroid hormone/RAR subfamily can be limited. COUP-TFII can also actively silence basal and activated transcription, likely through direct interaction with TFIIB or other general transcription factors [84]. Therefore, COUP-TFII may antagonize the functions of other hormones and, in turn, alter the cellular responses to multiple-hormone signaling pathways, and it can have profound effects on metabolic homeostasis. 

COUP-TF protein interacts with RXR*α* to form heterodimer [92]. Using RXR*α* cDNA probe, COUP-TFs were identified to bind to RAREs. The original COUP-TF-binding site in the ovalbumin gene is also a RARE that is repressed by COUP-TF [93]. On the other hand, RXR*α* and COUP-TFII can bind to the same DR1 element individually or in the heterodimeric form in an electrophoresis mobility shift assay (EMSA). Cotransfection of COUP-TFII and RXR*α* suppresses RXR-mediated activation of CRBPII promoter, which contains one RARE [92]. COUP-TFs are involved in the modulation of RAR- and RXR-mediated responses to retinoids during embryogenesis [84]. Recently, COUP-TFII has been identified as a low-affinity RA receptor. The ligand binding of COUP-TFII is in an autorepressed conformation. At high concentrations, RA is able to promote COUP-TFII to recruit coactivators and to activate a COUP-TF reporter construct [94]. These observations suggest a linkage between RA and COUP-TFII signaling pathways.

#### 1.4.5. PPAR*β*/*δ* for Mediating RA Responses

PPARs are members of nuclear receptor superfamily. They take part in the regulation of a variety of physiological functions such as cell differentiation, metabolism, and immune functions [95]. So far, three PPAR isoforms, PPAR*α*, PPAR*β*/*δ*, and PPAR*γ*, are identified and reported. The expression of PPAR*β*/*δ* is ubiquitous, which can be detected in a lot of cell types and tissues [96, 97]. In rats, PPAR*β*/*δ* mRNA and proteins are expressed ubiquitously and often coexpressed with other PPARs [98]. Similar expression profiles are observed in mice [99–101]. In mice, PPAR*β*/*δ* protein can be detected in a variety of tissues with high expression levels in the small intestine, skin, and liver [102]. The expression levels of PPAR*β*/*δ* mRNA and protein in the liver of rats fasted for 12 hours are reduced and increased upon refeeding for 4.5 hours [103]. The expression of low levels of human PPAR*β*/*δ* mRNA can be detected in the adipose tissues, the skeletal muscle, the liver, the kidney, and the intestines [104].

It has been shown that RA is a high-affinity ligand (nanomolar range) for PPAR*β*/*δ* activation in COS-7 cells [105]. RA-mediated activation of PPAR*β*/*δ* plays a role in cell proliferation [106]. In certain types of cells, RA is, respectively, delivered to RAR and PPAR*β*/*δ* by cytosol retinoic acid-binding protein II (CRABP-II) and fatty acid-binding protein 5 [107]. The activation of these two receptors by RA has opposite effects on cell growth. RA can activate PPAR*β*/*δ* in preadipocytes and mature adipocytes [108, 109]. RA treatment prevents obesity development in diet-induced obesity (DIO) *via* increase of lipolysis [108] and inhibition of adipocyte differentiation [109].

However, when human PPAR*β*/*δ* was stably over-expressed in human keratinocyte cells, no change in PPAR*β*/*δ* target gene expression could be observed after the treatment of RA [110]. Many studies have shown that endocannabinoids regulate the activity of PPARs *via* direct and indirect mechanisms [111]. The expression of endocannabinoid receptor type 1 (CB1) is reduced upon overexpression of PPAR*β*/*δ*, suggesting other pathways that PPAR*β*/*δ* is involved in [112].

**Figure 1:**
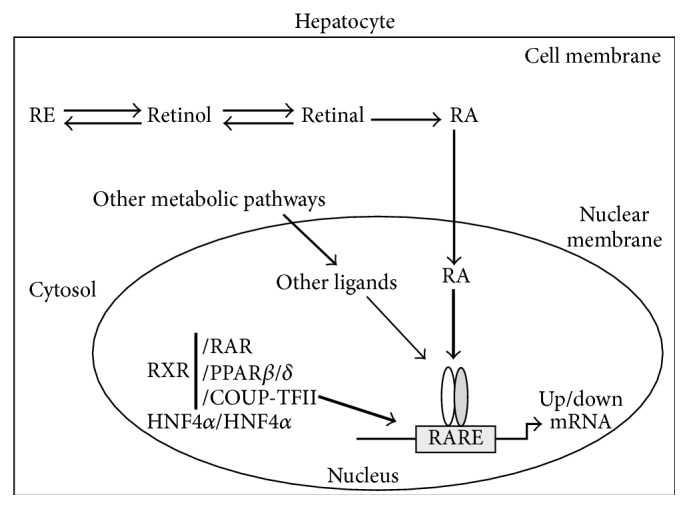
The effects of RA production and other metabolic factors on the activities of transcription factors associated with a retinoic acid-responsive element (RARE) in the promoter of an RA-targeted gene. In primary hepatocytes, RA is produced from retinal derived from retinol. Retinol can be esterified into retinyl ester (RE) or oxidized into retinal. The RARE can be occupied by retinoic acid receptor (RAR), retinoid X receptor (RXR), hepatocyte nuclear factor 4*α* (HNF4*α*), chicken ovalbumin upstream promoter-transcription factor II (COUP-TFII), and peroxisome proliferator-activated receptor *β*/*δ* (PPAR*β*/*δ*) in the forms of heterodimers or homodimers. These transcription factors receive signals from RA and metabolic pathways and regulate the expression of the RA-responsive gene.

 In summary, retinol in hepatocytes can be either stored as REs or oxidized into retinal as shown in Figure 1. RA is dynamically produced and sent to nucleus where it interacts with transcription factors associated with RARE in the enhancer regions of the RA-responsive genes. So far, the transcription factors that can be associated with a particular RARE include RAR, RXR, COUP-TFII, HNF4*α*, and PPAR*β*/*δ*. They form heterodimers or homodimers and bind to RARE to regulate the transcription rate of that gene. The nuclear receptors that occupy the RARE at any given moment may be determined by the VA status of the animal and the developmental, differentiation, and metabolic states of hepatocytes. These transcription factors integrate signals from RA and other metabolic pathways to control the gene expression positively and negatively. Other metabolic signals in combination with RA may affect the occupancy of the nuclear receptors bound to the RARE and their activations and may, in turn, alter the outcomes of the RA-mediated transcription responses.

## 2. VA's Roles in Glucose Metabolism

As a convenient energy source, glucose can be used by all cells that are in charge of different physiological functions. Glucose is metabolized differently in response to hormonal and nutritional statuses [113]. Hepatocytes utilize and produce glucose depending on the physiological needs and feeding status of a subject. These processes are regulated by nutritional and hormonal stimuli. VA status has been implied to play a role.

### 2.1. The Effects of VA Status on Hepatic Glycogen Contents

The roles of VA in glucose metabolism had been suggested. In 1937, the liver samples of patients who died of diabetes showed an elevation of VA contents [114]. When rats were fed a VA-deficient (VAD) diet to induce VA deficiency, their liver glycogen content was depleted [115]. Since VA deficiency also caused reduction of food intake in the VAD rats, a pair-feeding control group was included. The pair-fed rats with equal energy intake as that of VAD rats had higher hepatic glycogen content [115]. This depletion of hepatic glycogen content was attributed to the reduction of glycogenesis from trioses, rather than directly from glucose [115]. The phenomenon of the depletion of hepatic glycogen content in the liver of VAD rats has also been observed recently in my lab [116]. On the other hand, when rats were fed a diet with excessive amounts of VA for two days, an elevation of the hepatic glycogen content in the liver after fasting was observed [117]. It seems that hepatic VA content is directly correlated with the hepatic glycogen content.

### 2.2. The Effects of Animal VA Statuses and Retinoids on Hepatic *Gck* Expression

To initiate the hepatic glucose utilization, glucose is phosphorylated into glucose 6-phosphate by hexokinases in the first reaction of glycolysis. So far, four hexokinase isozymes, hexokinase I (A), II (B), III (C), and IV (D), have been identified. They have different tissue distributions, intracellular locations, and kinetic characteristics. Mammalian hexokinase IV (D) is also known as glucokinase (GK) (ATP: d-hexose 6-phosphotransferase, EC 2.7.1.1) [118–121]. Mutations of GK have been associated with MODY [122]. GK has two unique features that differentiate it from other hexokinase family members. It has low affinity for glucose (higher Km), and it is insensitive to allosteric inhibition mediated by physiological concentrations of glucose 6-phosphate, whereas other hexokinases are sensitive to it [118, 121, 123, 124]. In addition to glucose, other substrates of GK include fructose, mannose, and 2-deoxyglucose [118].

Generally, GK activity is mainly observed in the liver and pancreatic islets. The *β*-cells and liver GK isoforms have similar kinetic properties [125, 126]. In the short term, the GK activity can be regulated *via* allosteric interactions and covalent modifications such as binding to GK regulatory protein [127], phosphorylation by protein kinase A [128], and interaction with a cytosolic GK-associated phosphatase [129].

The long-term regulation of GK activity is done through the transcription of its gene, *Gck*, which is differentially regulated by an upstream promoter in pancreatic *β*-cells and a downstream promoter in hepatocytes [130, 131]. Using a strain of transgenic mice containing the cDNA of human growth hormone gene driven by an upstream *Gck* promoter fragment, *Gck* expression has been found in certain neuroendocrine cells of the pancreas, pituitary, brain, gut, thyroid, and lungs [132]. Activation of the upstream or downstream promoter leads to the generation of a *Gck* mRNA with a unique 5′ sequence derived from exon 1*β* or 1L, respectively. Since the translation initiation coden is located in the first exon, the GK proteins encoded by the different transcripts differ in the first 15 amino acids at the amino terminus [130, 131]. The mechanisms by which the upstream and the downstream promoters are selectively activated in different cells have not been revealed. The temporal and special control of the promoter selection must be determined by signals from differentiation and development processes.

The upstream and downstream promoters are activated based on the hormonal and nutritional conditions of the subject. For example, the hepatic, but not the pancreatic *β*-cell, *Gck* expression is reduced while fasting and increased while refeeding [130]. In the rat liver, insulin and glucagon, respectively, induce and inhibit the expression of *Gck* mRNA [133–135]. A long-standing question in the field is the insulin-responsive element (IRE) responsible for the insulin-induced hepatic *Gck* expression. Using the reporter gene constructs to study insulin-induced *Gck* promoter activation has not been successful. For example, a reporter gene construct containing a fragment of 5.5 kb hepatic *Gck* promoter failed to show any response to insulin treatment in rat primary hepatocytes [136]. This suggests that the IRE of the hepatic *Gck* might be in an area not included in this 5.5 kb promoter fragment. Alternatively, the unresponsiveness to insulin of the reporter gene constructs in the experimental settings could be due to the short-lived signal from insulin for *Gck *transcription as we have shown previously [137]. Interestingly, the hepatocyte-specific enhancer region that controls the activation of liver-specific promoter has been located in the region from −1003 to −707 relative to the transcription initiation site in primary rat hepatocytes. By contrast, this region acts as a silencer in FTO-2B hepatoma cells and has no effects on insulinoma cells [138]. Further studies are needed to identify the insulin-responsive element in the hepatic *Gck* promoter.

 The direct effects of RA on the expression of *Gck* mRNA in primary rat hepatocytes have been studied. Previously, others have shown that all-*trans* RA induced *Gck* expression in primary rat hepatocytes. However, nonadditive effect of RA on insulin-induced *Gck* expression was observed [139, 140]. Recently, we have shown that retinoids synergized with insulin to induce *Gck* expression in primary rat hepatocytes [137]. We have observed that all-*trans *retinol, retinal, and RA are able to synergize with insulin to induce *Gck *expression probably *via* the activation of RAR/RXR in primary rat hepatocytes. In addition, VA status also controls the expression of hepatic *Gck* expression [137]. The levels of *Gck* mRNA [116] and its activity [137] in the liver of VAD rats are lower than those of VA-sufficient (VAS) controls. In addition, a single treatment of RA increases the hepatic *Gck* expression in rats [137].

### 2.3. The Effects of Animal VA Status and Retinoids on *Pck1* Gene Expression

The plasma glucose homeostasis is achieved through its dietary intake and production from endogenous noncarbohydrate sources. During fasting, glucose is generated *via* gluconeogenesis. The cytosolic form of phosphoenolpyruvate carboxykinase (PEPCK-C, EC4.1.1.32), which catalyzes the conversion of oxaloacetate into phosphoenolpyruvate in the presence of GTP [141], has been considered to be the first rate-limiting enzyme of gluconeogenesis. The expression of PEPCK-C gene (*Pck1*) and its activity have been detected in the liver, kidney, adipose tissues, and some other tissues [141–143]. Other than gluconeogenesis, PEPCK-C activity has been considered to be responsible for glyceroneogenesis and cataplerosis [144]. Since no allosteric regulator of PEPCK-C has been identified, its activity is primarily controlled by the level of *Pck1* mRNA [141–143].

The transcription of hepatic* Pck1* is regulated by hormonal and nutritional stimuli. In the liver, fasting, diabetes, carbohydrate-free diets, and high-fat diets increase *Pck1* gene expression, whereas refeeding, insulin treatment, and high-carbohydrate diets decrease *Pck1* gene expression. Glucagon, glucocorticoids, thyroid hormone, and RA induce hepatic *Pck1* expression, while insulin and glucose inhibit it [145].

The transcription regulation of *Pck1* has been studied extensively [142, 143]. The protein-binding sites at the proximal region of rat *Pck1* promoter have been determined by DNase I footprinting assays [146, 147]. Multiple regulatory elements including distal proximal cyclic AMP-responsive element (CRE) [148] have been identified. Proteins in rat liver nuclear extracts can interact with the two CREs and six additional binding sites (P1 to 6) [142]. The transcription factors that bind to these sites include HNF4*α* [149], glucocorticoid receptor [148, 150–153], RAR [154–157], TR [154, 158], LXR [159], COUP-TF [149], the Forkhead family of transcription factors [160], C/EBP and cAMP regulatory element-binding (CREB) protein [161], and SREBP-1c [162, 163]. Additionally, transcription coactivators, such as CREB-binding protein (CBP) [164], steroid receptor coactivator 1 (SRC1) [165], and peroxisome proliferator-activated receptor gamma coactivator-1-alpha (PGC-1*α*) [166], also participate in the regulation of hepatic *Pck1* gene expression.

Using reporter gene assays and hepatoma cells, two RAREs have been identified in the hepatic *Pck1* promoter [154, 155, 167]. When PEPCK/bovine growth hormone (bGH) transgenic mouse strains are used, VA deficiency causes the reduction of the transgene's transcription [167]. The RARE1 is considered to be important for mediating the RA-regulated *Pck1* expression in the mouse liver [157]. In mice, VA status affects the association of RNA polymerase II and histone code of *Pck1* promoter [168, 169]. Later on, these RAREs have been shown to bind to HNF4*α*, RXR*α*, RAR*α*, PPAR*α*, and COUP-TFII [170].

In an attempt to study the lipophilic molecules that affect insulin-regulated gene expression in primary rat hepatocytes, we have obtained lipophilic extract (LE) from the rat liver. My original study has shown that this LE induces *Pck1* expression and attenuates insulin-mediated suppression of *Pck1* transcription in primary rat hepatocytes [171]. Interestingly, the same LE synergizes with insulin to induce *Gck* expression in the same cells [137]. The active molecules in the LE are retinol and retinal [137]. We have shown that retinol, retinal, and RA all can affect insulin-suppressed *Pck1* expression [172]. In rat primary hepatocytes, the proximal one of the two RAREs in the rat *Pck1* promoter [154–156] is responsible for mediating the retinoid effects [172]. The expression of hepatic *Pck1* mRNA in rats seems to respond to VA deficiency differently from that in mice. We have shown that the hepatic *Pck1* mRNA level in the Zucker lean (ZL) rats is not reduced in VA-deficient state [116]. VA deficiency only reduces the hepatic *Pck1* mRNA in the Zucker fatty (ZF) rats [116].

**Figure 2:**
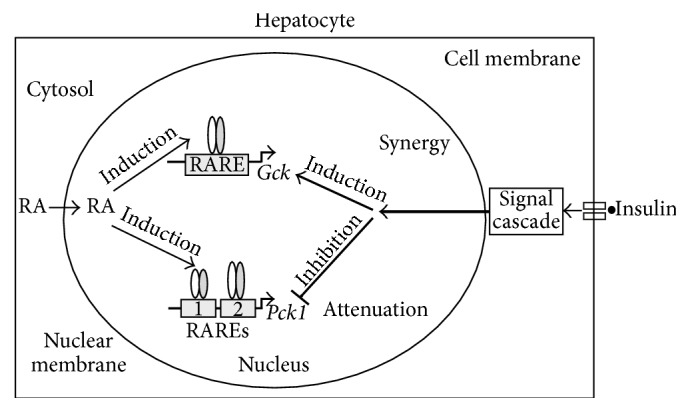
RA synergizes with insulin to induce *Gck* expression and attenuates insulin-suppressed *Pck1* expression. RA induces the expression levels of *Gck* and *Pck1* via the activation of both RAR/RXR (the oval dimmers on the RAREs) in the absence of insulin. Insulin alone stimulates the expression of *Gck* and suppresses the expression of *Pck1*. In the presence of both insulin and RA, the expression of *Gck* is further increased (synergy). On the other hand, the insulin-mediated suppression of *Pck1* expression is attenuated. This is because RA still induces *Pck1* expression *via* activation of RAR in the presence of insulin. Therefore, *Pck1* transcript level in the RA + insulin group is higher than that in the insulin group (attenuation).

It is important to note that the respective activities of GK and PEPCK-C are induced in different physiological conditions. For example, insulin induces *Gck* and suppresses *Pck1 *expression as shown in Figure 2. In primary rat hepatocytes, activation of either RARs or RXRs by RA is sufficient to induce the expression of both *Pck1* [172] and *Gck* [137]. However, activation of RAR potentiates (for the insulin-mediated induction of *Gck *expression) and attenuates (for the insulin-mediated suppression of *Pck1 *expression) insulin actions at the same time in the same hepatocytes. For* Gck*, RA-mediated activation of RARs and RXRs can be potentiated by insulin [137], which results in the synergy. For *Pck1*, only RA-activated RXRs, but not RARs, are inhibited by insulin, which results in the attenuation [172]. This causes the production of more *Pck1* mRNA in the presence of RA than in the absence of it even in the insulin-suppressed state. It indicates that the mechanisms of RA-mediated activation of RARs and RXRs probably depend on the promoter context or the isoforms associated with the promoters of their downstream genes in the presence of insulin. Therefore, the roles of retinoids in hepatic glucose metabolism deserve further investigation, especially the roles of the interactions between retinoids and insulin signaling pathways in the regulation of gene transcription. 

### 2.4. Role of RBP4 in Insulin Resistance

Plasma VA is bound to RBP4, which is mainly produced in the liver [13]. The *Rbp4 *mRNA is also expressed to a certain extent in adipose tissues and kidney [13]. In the plasma, retinol-RBP4 complex is associated with transthyretin (TTR), a process to increase the size of the holo-RBP4-TTR complex to reduce the loss during renal filtration [173].

Recently, the plasma RBP4 has been suggested to play roles in metabolic homeostasis. It has been reported that insulin-resistant human subjects and animals have higher plasma RBP4 levels than normal ones due to its elevated production from adipose tissues [174]. In mice, RBP4 administration has been shown to reduce insulin sensitivity and increase the expression levels of hepatic gluconeogenic genes, such as *Pck1* and the catalytic subunit of glucose 6-phosphatase (*G6pc*) [175]. Additionally, reduction of plasma retinol and RBP levels has been reported in type 1 diabetic patients [176] and in streptozotocin- (STZ-) induced diabetic rats [177]. *Rbp4* knockout mice have improved insulin sensitivity [175]. The treatment fenretinide, a synthetic retinoid derivative that disrupts the association of RBP4 and TTR, improves insulin sensitivity in HFD-induced obese mice [175].

When a compound specifically developed to disrupt the RBP4 and TTR interaction was used to treat mice, it successfully disrupted the formation of RBP4-TTR complex and caused the reduction of plasma RBP4 level [178]. However, the reduction of plasma RBP4 level did not result in an improvement of insulin sensitivity [178]. In addition, the improvement of insulin sensitivity in *Rbp4 *knockout mice could not be observed in this study [178]. Therefore, the elevation of insulin sensitivity in mice treated with fenretinide is probably due to a mechanism other than the disruption of RBP-TTR complex and reduction of plasma RBP4 levels.

## 3. Roles of VA in FA and TG Metabolism

FAs are needed for many aspects of cellular functions from signal transduction to energy production. The continuous survival of a given cell depends on the constant energy supplies from the environment or storage sources. The storage of FA in the form of TG ensures the constant energy supply in a cell or organism for a prolonged period of time when environment energy is limited. In animals, the homeostasis of FA and TG is dynamically regulated by the liver and the adipose tissues in response to nutritional and hormonal stimuli [179, 180]. During fasting or starvation, FAs are released from adipose depots after lipolysis and oxidized in the liver and muscle for energy production [181]. After refeeding, dietary and newly synthesized FAs are converted to TGs for the storage in the adipose tissues [182]. On the other hand, the altered regulation of this system may cause problems. For example, the excessive synthesis and storage of FAs in the form of TGs can cause the development of obesity and other metabolic diseases. 

### 3.1. The Effects of VA Status on the Body Fat and Plasma Lipid Profile

VA was first recognized as a dietary lipophilic factor which was essential to support the normal growth of lab animals [3, 4]. Subsequently, it was observed that, along with the reduction of body mass, the VAD rats that had been fed a VAD diet at weaning (21 days old) for more than 9 weeks had reduction of carcass fat, but not cholesterol content [183]. On the other hand, this reduction of body fat was not observed in the rats of the pair-feeding group, which were given the same amount of a VAS diet in weight and calories as the one consumed by the VAD rats [183]. These results indicate that the reduction of food intake alone could not be used to explain the depletion of body fat in the VAD rats.

The VA status also seems to affect the hepatic expression of apo AI mRNA. The expression of hepatic apo AI has been shown to be sensitive to VA status [184]. When the Lewis strain rats develop VA deficiency, the expression level of hepatic, but not intestinal, apo AI mRNA is elevated. This induction is dropped upon single dosage of RA treatment [184]. On the other hand, in the intestine and liver of VA-deficient Wistar rats, both all-*trans* RA and 9-*cis* RA can induce apo AI mRNA levels [185]. The same treatment can only induce the mRNA level of apo CIII in the intestine, but not in the liver [185]. In humans, the plasma VA levels are positively correlated with the elevations of proteins of the AI-CIII-AIV gene cluster in familial combined hyperlipidemia patients [186].

When male Wistar rats were fed a VAD diet for 3 months after weaning (21 days old), they had lower plasma TGs and cholesterol levels than the control ones fed a VAS diet [187]. When the hepatic synthesis of FAs and phospholipids was analyzed in the liver slices, the incorporations of [^14^C]choline into phosphatidylcholine and [^14^C]acetate into FAs were also reduced in the VAD rats compared with their VAS controls [187]. The VAD rats had lower activities and mRNA levels of acetyl-CoA carboxylase, but higher activity and mRNA level of carnitine palmitoyltransferase-I than VAS rats had [187]. These results demonstrate that there is a reduction of hepatic fatty acid synthesis along with an induction of hepatic fatty acid oxidation in the liver of VAD rats.

My lab has conducted experiments and shown that VA status affects the obesity development in ZF rats and controls plasma TG levels in ZL and ZF rats [116]. ZF rats, a rat model of obesity development [188], develop obesity due to a missense mutation in the extracellular domain of all leptin receptor isoforms [189–191]. They have been used for the studies of obesity, insulin resistance, and other aspects of metabolic diseases [192, 193]. We have analyzed growth, plasma parameters, and expression levels of hepatic genes of male ZL and ZF rats fed a VAD or a VAS diet at weaning (21 days of age) for 8 weeks. Body masses of ZL and ZF rats fed the VAD diet are lower than those of their corresponding controls fed the VAS diet at the end of the feeding period. The VAD ZL or ZF rats begin to take less food than the VAS ones after five weeks on the diet. The VAD ZL or ZF rats have lower plasma glucose, TG, insulin, and leptin levels than their corresponding VAS rats have. The liver glycogen content, net weights of epididymal fat and liver, and fat-to-body mass ratio (ZL only) in the VAD rats are also lower than those in the VAS ones [116]. This demonstrates that VA status affects plasma TG levels in both ZF and ZL rats and the obesity development in ZF rats. This phenomenon may be caused by the combination of VA deficiency and hypoinsulinemia since insulin secretion is impaired in the VAD rats [194].

### 3.2. The Development of Hypertriglyceridemia in Patients Receiving Retinoic Acid-Based Treatments

#### 3.2.1. The Uses of RA-Based Medicines in Clinic

RA-based medicines, tretinoin (all-*trans* RA), isotretinoin (13-*cis* RA), and alitretinoin (9-*cis* RA), have been used in clinic to treat diseases, such as acute promyelocytic leukemia and skin disorders [11]. In fact, isotretinoin has been widely used to treat severe acne. Early clinical observations indicate that the medical use of isotretinoin in human subjects results in the elevation of their plasma TG levels [195–197]. A significant portion of patients with acne receiving isotretinoin treatment developed hypertriglyceridemia [9, 10]. In addition, the systemic administration of isotretinoin in patients with acne resulted in the elevation of plasma levels of alanine aminotransferase (ALT) and aspartate aminotransferase (AST), indicating the liver damage [198]. The hypertriglyceridemia is also associated with reduced insulin sensitivity and elevated TG in the very-low-density lipoprotein (VLDL) fraction [195]. The treatment of all*-trans* RA resulted in body mass gain and hypertriglyceridemia in patients with acute promyelocytic leukemia [199, 200]. Moreover, RA treatment also raises plasma TG levels in rats [201–203].

#### 3.2.2. The Mechanism of RA-Mediated Hypertriglyceridemia in Patients

Apo CIII is produced from liver and considered an inhibitor of LPL activity [204]. Mice with *Apoc3* gene knockout have hypotriglyceridemia and enhanced in vivo VLDL-TG clearance [205]. On the other hand, transgenic mice with overexpression of apo CIII develop hypertriglyceridemia [206]. The RA-induced hypertriglyceridemia has been attributed to RA-induced apo CIII expression.

In healthy human subjects, isotretinoin treatment causes a rise of plasma apo CIII protein level [207]. This is caused by the induction of *Apoc3* mRNA *via* the activation of RXR at a RARE in its promoter [207]. The plasma TG levels in rats treated with RXR-specific agonist (LG100268) are induced rapidly and constantly [208]. This results in the reduced activity of LPL in the heart and skeletal muscle of those animals [208]. The RARE is identified in the C3P region which controls the hepatic specific expression of *Apoc3* gene [75]. Interestingly, the same site is also considered to be responsible for mediating the effects of HNF4*α* and COUP-TFII on *Apoc3* gene expression [209]. The expression of *Apoc3* seems to be regulated differently by the C3P-binding proteins [75].

It is worth to note that insulin suppresses *Apoc3 *mRNA expression in STZ-induced diabetic mice [210]. In addition, the activation of RXR by LG100268 treatment induces insulin sensitivity and reduces TG levels in obese and diabetic mice [211]. The understanding of the regulatory mechanism that integrates hormonal and nutritional signals at the regulatory sites such as C3P in *Apoc3* promoter will shed some lights on the regulation of hepatic lipid homeostasis. It will also provide a novel target for the treatment of the RA-induced hypertriglyeridemia.

#### 3.2.3. The Effects of RA Treatment on Obesity Development in Mice

Interestingly, the RA administration in rodents causes reduction of body mass as summarized in [212]. This kind of reduction of body mass has been associated with enhancement of insulin sensitivity in genetic [213] or DIO mice [108]. The mechanisms of RA-mediated attenuation of insulin resistance have been thought to be the induction of energy expenditure through PPAR*β*/*δ* activation [108] and the inhibition of adipogenesis through RXR*γ* activation [109]. On the other hand, retinol can be metabolized in adipose tissues [214]. The mice with deletion of *Raldh1 *gene (*Raldh1*−/−) have elevated retinal in their adipose tissues and are resistant to diet-induced obesity and insulin resistance [26]. This has been attributed to the suppression of adipogenesis by retinal-inhibited activation of PPAR*γ* and RXR [26]. It is interesting to note that either retinal or RA can inhibit the adipogenesis and cause the improvement of insulin sensitivity in mice based on these studies.

### 3.3. Roles of VA and Retinoids in the Regulation of Hepatic Lipogenic Gene Expression

Insulin resistance, diabetes, and other metabolic abnormalities are associated with profound changes of hepatic lipid and glucose metabolism. This can be attributed, at least in part, to the altered expression of genes involved in glucose and lipid metabolism [215]. The hepatic lipogenesis contributes to the homeostasis of FAs and TGs.

#### 3.3.1. The Regulation of Hepatic *Srebp-1c* Expression by VA Status and Retinoids Treatment

Insulin plays an essential role in the hepatic FA synthesis. It has been known for a while that insulin is needed for the glucose usage for lipogenesis and CO_2_ production in the liver [216]. The hepatic FA synthesis rises sharply and continuously after rats are fasted and then refed a high-carbohydrate diet [217]. The amount of fatty acid synthase (FAS) is induced in normal rats refed a high-carbohydrate diet after fasting and in STZ-induced diabetic rats after insulin administration [218]. In the insulin-deficient rats, the induction of FAS after refeeding occurs only when insulin injection is injected [219], a process attributed to the induction of FAS gene (*Fas*) [220, 221].

The transcription of hepatic *Fas* is controlled by a transcription factor named SREBP-1c [222]. In the liver-specific *Srebp-1c* knockout mouse liver, the induction of *Fas* and other lipogenic genes by refeeding after fasting is blunted [223]. Interestingly, insulin also specifically induces the* Srebp-1c* expression in the liver [224] *via* two liver X receptor elements (LXREs) and one sterol-responsive element (SRE) [225]. However, in liver-specific insulin receptor knockout mice, the induction of hepatic *Srebp-1c* by refeeding largely remained unchanged [226]. This suggests that nutrient-derived signals may activate downstream of insulin receptor signaling pathway to regulate *Srebp-1c* expression and hepatic lipogenesis [226].

 As stated in the previous section, my research group has observed that the VAD ZL and ZF rats have lower plasma TG levels than their VAS controls [116]. This is accompanied by the reduction of the hepatic expression of *Srebp-1c* mRNA in VA-deficient animals [116]. My research group has reported for the first time that retinoids synergize with insulin to induce the expression of *Srebp-1c* in primary rat hepatocytes [227]. Further analysis of the *Srebp-1c* promoter reveals that the previous identified LXREs responsible for mediating insulin-induced *Srebp-1c* expression are also RAREs [227]. These observations show that retinoids (nutritional) and insulin (hormonal) signals converge to regulate the FA synthesis in the liver.

#### 3.3.2. The Effects of VA Catabolism on the Regulation of *Srebp-1c* Expression in Primary Rat Hepatocytes

More recently, retinoids have been proposed to play roles in glucose and lipid metabolism and to maintain energy homeostasis [228, 229]. VA (retinol) is reversibly converted into retinal, and retinal is irreversibly converted into RA [23]. Retinal, the precursor of RA production, is able to synergize with insulin to induce the *Srebp-1c* expression in primary rat hepatocytes [227]. This suggests that the dynamic production of RA influences the expression of hepatic lipogenic genes. It has led us to investigate the roles of RA production in the insulin-resistant animals.

In the liver of the *Raldh1*−/− mice, RA production is reduced in response to a load of retinol [230]. The *Raldh1*−/− mice are resistant to the development of obesity and insulin resistance after being fed an HFD [26]. This is attributed to the elevation of retinal content due to the lack of conversion of retinal to RA in the adipose tissues of the* Raldh1*−/− mice. Additionally, the liver of the *Raldh1*−/− mice has altered regulation of the genes for gluconeogenesis and significantly attenuated hepatic TG synthesis, indicating the important role of hepatic VA metabolism in glucose and lipid homeostasis [231].

The hepatic expression levels of RALDH1 and RALDH2 are induced in mice fed a high-cholesterol diet [232]. SREs are located in the promoters of these two genes, which are induced by SREBP-1c overexpression. The expression of *Srebp-1c* is induced by the oxysterols derived from the high-cholesterol diet [232]. This research work demonstrates the interaction of cholesterol and RA metabolism in the liver. In addition, it has been shown that the level of *Raldh1* mRNA is elevated in the kidney of *db/db* mice [233]. Both *db/db* mice and ZF rats develop obesity due to the mutation of leptin receptor [234].

In an attempt to understand the roles of VA metabolism in the states of metabolic diseases, we have compared the expression levels of enzymes responsible for the production of retinal and RA in the freshly isolated and cultured primary hepatocytes from ZL rats and ZF rats [45]. We have found that, in the primary rat hepatocytes, the enzymes responsible for the conversions of retinol to retinal and from retinal to RA are most likely RDH2 and RALDH1, respectively. The expression levels of *Raldh1* mRNA and its protein are higher in hepatocytes from ZF rats than in those from ZL rats [45]. When overexpressed, RALDH1 introduces the retinal-mediated induction of *Srebp-1c* in INS-1 cells, which do not respond to retinal in our previous study [227]. These results indicate that insulin-resistant animals such as ZF rats may have alterations in retinoids metabolism.

Thus, we have hypothesized that the change of RA production from the oversupply of dietary VA due to the hyperphagia of ZF rats results in higher *Srebp-1c* expression in ZF hepatocytes [45]. The elevated SREBP-1c expression can further induce *Raldh1* expression to create a feed-forward mechanism that could be one of the reasons responsible for the increased lipogenesis in the liver of ZF rats. All of these observations indicate the potential roles of RA metabolism in the development of obesity and insulin resistance, which deserves to be further studied.

## 4. VA's Roles in Mediating Hepatic Insulin Action and Insulin Resistance

### 4.1. Insulin and Its Signal Transduction

The insulin molecule is made of two chains linked by one intra- and one interchain disulfide bonds [235]. The insulin receptor is a transmembrane and heterotetramer tyrosine kinase receptor (two *α* and two *β* subunits) linked by disulfide bonds as well [236]. The binding of insulin to its receptor causes conformation changes and transphosphorylation of the *β*-subunit tyrosine kinase [237, 238], which initiates the signal transduction [239]. Protein-protein interactions are involved in the transduction of insulin signal. The signal is transduced by multiple components in a complex network containing cascades of kinases and phosphatases [240, 241]. The first intracellular components mediating insulin signals are insulin receptor substrates (IRSs), protein molecules phosphorylated by insulin receptor *β*-subunit and mediating insulin signals [242].

The insulin signal event is further amplified by phosphatidylinositide 3-kinases (PI3K)/protein kinase B (PKB/Akt) pathway and GRB2/mitogen-activated protein kinase (MAPK) pathway [243–245]. Another serine/threonine kinase that can be activated by PI3K is the atypical protein kinase C [246]. Human liver expresses several protein-tyrosine phosphatases (PTPs) [247]. Okadai acid, an inhibitor of protein phosphatase type 2A and type 1, inhibits glycogen synthesis and insulin-stimulated* Gck* expression in primary rat hepatocytes, indicating that dephosphorylation mechanism may also participate in mediating insulin signaling [248]. These components are shared by insulin-like growth factor (IGF) receptor and involved in mediating insulin and IGF signals [249].

### 4.2. Metabolic Diseases and Insulin Resistance

The rise of the number of people with one or more metabolic diseases such as obesity and diabetes in the world has become a public health problem [250]. The interactions of genetic and environmental/nutritional factors may be responsible for the current rise of metabolic diseases. The long evolutionary process has equipped human and animal bodies with a variety of regulatory mechanisms controlling these interactions [251, 252]. Human diets contain not only energy derived from macronutrients but also micronutrients and other factors, which may influence the metabolic outcomes. The roles of individual micronutrients in the development of metabolic diseases are still unrevealed.

One common property of human obesity and NIDDM is insulin resistance, in which a given amount of insulin produces less than normal physiological responses. Dietary factors and manipulations have been considered to play a role in the development of insulin resistance in humans and animals. For example, feeding an HFD to young and insulin-sensitive subjects for two days causes the development of glucose intolerance [253]. In the skeletal muscle of rats fed an HFD for 8 weeks, the insulin-induced glucose uptake is impaired [254]. FFAs have been considered the factors responsible for HFD-induced leptin and insulin resistance [255–257].

### 4.3. Hepatic Insulin Resistance and Vicious Cycle

The liver plays an essential role in the control of glucose and lipid homeostasis. It coordinately regulates the processes in responses to nutritional and hormonal stimuli. Insulin plays a major role in directing the liver's metabolic status [258], which is partially attributed to the regulation of the expression of genes involved in hepatic glucose and lipid metabolism [215, 259].

Insulin controls the expression of a variety of genes involved in glycolysis, glycogenesis and lipogenesis, and gluconeogenesis in the liver [145]. Generally, hepatic lipid and glucose metabolism is altered with the development of insulin resistance in obesity and NIDDM [258]. This can be reflected in the expression of insulin-regulated hepatic genes for glucose and FA metabolism. When the liver is insulin sensitive, insulin stimulates lipogenesis and inhibits gluconeogenesis. For example, to regulate hepatic glucose homeostasis, insulin increases the expression of *Gck* gene [119, 120] and *Srebp-1c* [224], a family member of SREBPs which regulate the hepatic cholesterol and FA biosynthesis, and their homeostasis [260]. It suppresses the expression of *Pck1* [142] and *G6pc* [145], which control the first and last steps of gluconeogenesis, respectively. When the liver is insulin resistant, insulin no longer inhibits gluconeogenesis. However, the liver still keeps the elevated lipogenesis. The elevated FA biosynthesis in the insulin-resistant state further facilitates insulin secretion from pancreatic *β*-cells, which probably enhances lipogenesis and results in a vicious cycle [113, 261]. The coexistence of the hepatic insulin resistance and sensitivity at the gene expression level has been observed in rodent obese and diabetic models [215, 258]. However, the mechanism of this coexistence of insulin sensitivity and resistance has not been revealed [261].

Recently, the SREBP cleavage-activating protein gene (*Scap*), an escort protein necessary for generating nuclear forms of SREBPs, has been knocked out in insulin-resistant *ob/ob* mice [262]. Deficiency of hepatic *Scap* reverses liver lipogenesis and steatosis in *ob/ob* mice. However, the plasma levels of glucose and insulin and the hepatic expression levels of *Pck1 *and *G6pc* were not different between the control *ob/ob* mice and the *ob/ob* mice with liver-specific *Scap* knockout [262]. It seems that the suppression of hepatic FA synthesis is not sufficient to correct insulin resistance. Whether other factors play a role in the hepatic insulin resistance is still an open question.

The clinical use of RA-based medicine such as isotretinoin (13-*cis* RA) caused elevation of plasma TG levels in human subjects [195–197]. This increase in plasma TG levels is also observed in rats [201–203]. From our VA status studies, we have shown that VA status affected the obesity development in ZF rats and reduced plasma insulin and TG levels in ZL and ZF rats, suggesting improvement of insulin sensitivity [116]. The hepatic expression of *Srebp-1c* was reduced in VA-deficient animals [116]. RA synergizes with insulin to induce the expression of *Srebp-1c *in primary rat hepatocytes [227]. On the other hand, RA treatment induces *Pck1 *expression and attenuates insulin-mediated suppression of its expression in primary rat hepatocytes [172]. All of these observations demonstrate that RA attenuates insulin action (insulin-suppressed *Pck1* expression) and still promotes insulin action (insulin-induced *Srebp-1c* expression) in the same primary hepatocytes.

### 4.4. Possible Mechanism by Which RA Production Causes the Development of the Vicious Cycle in Hepatocytes

**Figure 3:**
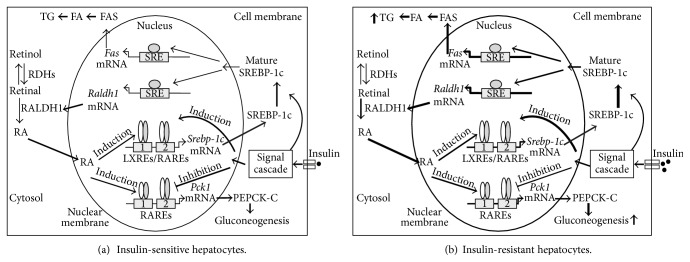
The effects of RA on the insulin-regulated *Srebp-1c* and *Pck1* expression levels, the protein levels of SREBP-1c, the expression levels of *Raldh1* and *Fas*, and the fatty acid synthesis and gluconeogenesis in insulin-sensitive (a) and insulin-resistant (b) hepatocytes. Please see the text in this paper for description. Up arrows next to the text indicate induction. The intensified weight of the lines indicates the induction of that part of the pathway.

Could RA or other metabolites from VA catabolism be a factor that contributes to the hepatic insulin resistance and the vicious cycle? Using the regulation of *Pck1* and *Srebp-1c* as the model, I have come up with the following scheme to show that retinoid metabolism could be a factor that causes the vicious cycle in the liver. As shown in Figure 3(a), in insulin-sensitive hepatocytes, RA derived from retinal coordinates with insulin to regulate the expression of *Srebp-1c* and *Pck1.* The SREBP-1c protein is processed to mature form, a step that can be facilitated by insulin [263]. The mature form of SREBP-1c enters into the nucleus and induces the mRNA levels of *Raldh1* and *Fas*, which are, respectively, transcribed into RALDH1 and FAS. RALDH1 catalyzes the conversion of retinal to RA. FAS catalyzes the synthesis of palmitate, which is esterified into TGs or elongated to longer FAs. The *Pck1* mRNA is used to translate into PEPCK-C protein, which contributes to glucogenogenesis. In the insulin-resistant hepatocytes as shown in Figure 3(b), the elevation of *Raldh1* expression due to leptin deficiency [45] or induction of SREBP-1c by high-cholesterol feeding [232] induces the production of RA. The elevated RA levels will stimulate the transcription of *Srebp-1c* and *Pck1*. This leads to more production of SREBP-1c, and its mature form, in the presence of higher insulin levels in the insulin-resistant state. The induction of SREBP-1c further enhances the transcription of *Raldh1* and *Fas*, and their corresponding proteins for the production of more RAs and FAs, respectively. The RA production and SREBP-1c expression form a feed-forward cycle, which causes more TG and RA production in the insulin-resistant liver. At the same time, more of the PEPCK-C protein is generated due to the elevated *Pck1* transcription by RA stimulation, which is not responsive to insulin inhibition as we have shown in [172]. This leads to elevated gluconeogenesis in the insulin-resistant liver. Eventually, the phenotypes appear as if insulin no longer suppresses gluconeogenesis while it still stimulates FA synthesis in the insulin-resistant state.

## 5. Perspectives and Future Directions

I have proposed a mechanism by which VA metabolism contributes to the development of hepatic insulin resistance. It explains some observations reported in the previous studies of rodent obese and diabetic models [215, 258]. However, it leaves more open questions. The first one is what the initiation factor is. So far, the elevated expression of *Raldh1* mRNA in the liver has been observed in ZF rats [45] and mice fed a high-cholesterol diet [232]. These two conditions cause a lot of physiological changes, such as overfeeding in ZF rats and change of cholesterol biosynthesis in response to the feeding of high-cholesterol diet. Knocking down *Raldh1* specifically in hepatocytes is needed to answer these questions. The second question is whether RA synthesis in hepatocytes is a regulated process in response to dietary or hormonal stimulations. If *Raldh1* is a downstream gene of SREBP-1c, it should be regulated when the SREBP-1c level changes. As the expression of *Srebp-1c* responds to insulin stimulation and oxysterols, the regulation of *Raldh1* expression in the liver should be studied. The third question is why it is RALDH1, but not other RALDHs. Specific inhibitors of RALDHs may be needed to answer this question. Last question is whether we can attenuate insulin resistance by suppressing the RALDH1 activity in the liver. It has been shown that *Raldh1*−/− mice are still insulin sensitive when they are on an HFD [26]. This has been attributed to the effects on the adipogenesis. However, the liver of those knockout mice has reduced lipid synthesis [231]. Further studies are warranted to answer all of these questions. Hopefully, it can become an intervention point for the treatment of insulin resistance and metabolic diseases.
